# A cross-sectoral approach to utilizing health claims data for quality assurance in medical rehabilitation: study protocol of a combined prospective longitudinal and retrospective cohort study

**DOI:** 10.1186/s12913-023-10074-w

**Published:** 2023-10-17

**Authors:** Vanessa Kaiser, Urs A. Fichtner, Caroline Schmuker, Christian Günster, Diana Rau, Lena Staab, Erik Farin-Glattacker

**Affiliations:** 1 Section of Health Care Research and Rehabilitation Research, Institute of Medical Biometry and Statistics, Faculty of Medicine, Medical Center, University of Freiburg, Freiburg, Germany; 2 AOK Research Institute (WIdO), Berlin, Germany

**Keywords:** Quality assurance, Quality of care, Medical Rehabilitation, Cross-sectoral quality assurance, Claims data, Health insurance, Risk-adjustment model

## Abstract

**Background:**

Measuring the quality of provided healthcare presents many challenges, especially in the context of medical rehabilitation. Rehabilitation is based on a holistic biopsychosocial model of health that includes a person’s long-term functioning; hence, outcome domains are very diverse. In Germany, rehabilitation outcomes are currently assessed via patient and physician surveys. Health insurance claims data has the potential to simplify current quality assurance procedures in Germany, since its comprehensive collection is federally mandated from every healthcare provider. By using a cross-sectoral approach, quality assessments in rehabilitation can be adjusted for the quality provided in previous sectors and individual patient risk factors.

**Methods:**

SEQUAR combines two studies: In a prospective longitudinal study, 600 orthopedic rehabilitation patients and their physicians are surveyed at 4 and 2 time points, respectively, throughout rehabilitation and a follow-up period of 6 months. The questionnaires include validated instruments used in the current best-practice quality assurance procedures. In a retrospective cohort study, a nationwide claims database with more than 312,000 orthopedic rehabilitation patients will be used to perform exploratory analysis for the identification of quality indicators. The identified SEQUAR claims data quality indicators will be calculated for our prospective study participants and tested for their ability to approximate or replace the currently used, best-practice quality indicators based on primary data.

**Discussion:**

The identified SEQUAR quality indicators will be used to draft a novel, state-of-the-art quality assurance procedure that reduces the administrative burden of current procedures. Further research into the applicability to other indications of rehabilitation is required.

**Trial registration:**

WHO UTN: U1111-1276-7141; DRKS-ID: DRKS00028747 (Date of Registration in DRKS: 2022/08/10).

**Supplementary Information:**

The online version contains supplementary material available at 10.1186/s12913-023-10074-w.

## Background

Roughly 100,000 avoidable deaths per year in US hospitals occur due to medical error—that is the imposing figure the Institute of Medicine reported in 2000 [1]. The very influential report, aptly titled “To Err is Human”, has been credited as a turning point for patient safety and quality control in healthcare [2, 3]. In Germany, efforts to implement systems of quality control in healthcare (also known as quality assurance) are considerably older—lawmakers codified federal mandates to monitor, assess, and improve the quality of care in hospitals, doctor’s offices and other medical facilities in 1988 via internal management systems and external assessment procedures [4]. However, measuring the quality or comprehensive, long-term outcomes of medical care was (and still is) not an easy feat. Patient surveys have to be conducted and processed, medical records examined and coded, charts reviewed and abstracted.

### Quality assurance with claims and administrative data

To mitigate the efforts of primary data collection, researchers have been tapping into the potential of administrative or health insurance claims data. This (often billing) data is routinely collected, readily available, and has been successfully used in a variety of healthcare and public health research: Estimating disease-specific cost to a healthcare system [5], comparing healthcare costs across different countries [6], or tracking opioid use in a population over time [7], to name a few examples.

Some countries use administrative and claims data to publish nationwide, comprehensive quality of care ratings for their hospitals or even individual doctors (e.g., “Hospital Compare” in the US, or the Care Quality Commission’s website in the UK). In others, administrative and claims data has been used to develop quality indicators, which are standardized measures that quantify the rather abstract concept of care quality in numbers (e.g., staffing ratios, post-op mortality rates, average wait times; [8]). Quality indicators can be disease-specific, like Japan’s nationwide quality of cancer care quality indicators [9]), or a general assessment of hospital quality, like AHRQI™ in the US (Agency for Healthcare Research and Quality Indicators; [10]) and QSR in Germany (Quality Assurance with Routine/Claims Data, German: Qualitätssicherung mit Routinedaten; [11]). Claims data has also been used to assess the proportion of patients participating in cardiac rehabilitation after a heart attack [12], approximate the quality of care in rehabilitation facilities after knee replacement [13], and evaluate the effect of orthopedic rehabilitation after hip fractures on hospital readmission [14].

### Quality of care in medical rehabilitation

Measuring quality of care and long-term outcomes of medical rehabilitation tends to be particularly difficult because outcome domains are so diverse. Medical rehabilitation and its intended outcomes are based on the biopsychosocial model of WHO’s Classification of Functioning, Disability and Health (ICF): Illness, health, and a person’s function are a result of a complex interaction between the person and their environment, influenced by biological factors (like physical health), psychological factors (like coping skills), and social factors (like support networks; [15]). Intended outcomes include restoring function and well-being in many areas of a patient’s life, such as a return to regular daily activities, family life, and the workforce [16].

In much of the world, medical rehabilitation is provided in outpatient centers, part time, with limited therapies during evening or weekend hours [17]. Germany (like Austria and Switzerland), however, administer medical rehabilitation mostly in inpatient rehabilitation facilities (IRFs). These IRFs can be traced back to a century’s old tradition of “treating” a wide range of diseases in health resorts and sanatoriums with natural and holistic remedies such as fresh air, water treading, and thermal baths [18, 19]. Today, the average patient completes German inpatient rehabilitation in 25 days [20] and receives therapies and trainings that target all domains of life in accordance with the ICF model. Medical rehabilitation is an integral part of the German healthcare system—in 2019^1^, the biggest German insurance fund DRV spent roughly 7.5 billion US Dollars on medical rehabilitation [21].

### Rehabilitation quality assurance in Germany

These intricacies of German medical rehabilitation make external procedures of quality assurance in IRFs a very elaborate process. One of the two widely used existing procedures, QS-Reha® (Quality Assurance in Rehabilitation®), requires patients and physicians to participate in an empirical survey [22, 23]. Take rehabilitation for musculoskeletal indications, for example: Patients are to complete two questionnaires distributed to them by the IRF at two set time points, while doctors submit one report form. That amounts to 35 pages of primary data per patient that have to be collected and submitted to the evaluating party, who has to process and record them into databases. Conservatively, 55,000 musculoskeletal rehabilitation patients participated in the last wave of evaluations, which means that 2 million pages of primary data had to be handled [23]. This places an enormous amount of administrative burden on IRFs that are already struggling with understaffing [20, 24].

Because Germany’s healthcare system is predicated on (mandatory) statutory health insurance (SHI), available claims data is very comprehensive. Aside from personal data (names, addresses, dates of birth), German SHIs record billing and diagnostic data on inpatient stays; outpatient treatment; pharmacological treatment; physical, occupational and speech therapy; medical aids like walkers, hearing aids, and prescription glasses; and any additional nursing care or nursing services received in-home. The collection and transfer of this data from medical providers to SHIs is mandated by the German Social Code (e.g., parts of Book V and X), which also allows for processing of claims data for research purposes [25]. It seems that German claims data would be uniquely suited to employ in procedures of quality assurance, yet we know very little about its potential in the context of medical rehabilitation.

Additionally, there is very limited research examining the impact of quality of care received in a previous sector (e.g., an operating hospital before follow-up rehabilitation) on the quality and long-term outcomes of rehabilitation, even though many researchers (and lawmakers) have identified the need for German healthcare research to coordinate across sectors [20, 26, 27]. It does stand to reason that the treatment outcome of orthopedic rehabilitation after joint replacement hinges on the success of the previously performed surgery—patients that received surgery and postoperative care in a high-quality hospital are likely in better health at the start of follow-up rehabilitation (baseline). Thus, adjusting for risks in previous sectors (e.g., hospital quality, perioperative complications, pre-existing conditions) could likely improve the accuracy of quality assessment in subsequent sectors.

### Research aims

In SEQUAR (Cross-**Se**ctoral **Qu**ality **A**ssurance With **R**outine/Claims Data), we will close the gap in research by utilizing cross-sectoral claims data from a major German SHI, AOK. We will evaluate its potential to simplify current quality assurance procedures in medical rehabilitation and improve risk adjustment modelling, which accounts for individual patient risk factors when determining a facility’s quality. Available claims data (CD) will be analyzed to identify CD outcome variables (outcomes on patient level, e.g.: pain medication prescription after discharge) and CD quality indicators (outcomes on facility level, e.g.: proportion of patients that are prescribed pain medication after discharge), and evaluate their proximity to outcomes we obtained via primary data collection (PD outcomes; e.g.: patient-reported rating of pain).

#### Hypotheses

Using a sample of primary knee or hip replacement patients in orthopedic follow-up rehabilitation, we will test the following hypotheses:

H1a: Patient-level outcomes (outcome variables) based on claims data (CD outcome variables) show high association with patient-level outcomes as measured by a primary data quality assurance protocol currently considered best practice (PD outcome variables).

H1b: Similarly, facility-level outcomes (quality indicators) based on cross-sectoral SHI claims data (CD quality indicators) are correlated with facility-level outcomes based on the current best-practice quality assurance protocol collecting primary data (PD quality indicators).

H2: A risk-adjustment model for rehabilitation outcomes as measured by PD outcome variables improves when utilizing risk factors based on cross-sectoral SHI claims data (risk factors on patient- and facility levels) compared to a risk-adjustment model without CD risk factors.

## Study design and methods

SEQUAR combines two studies in one project: A prospective longitudinal multicenter study collecting patient- and health professional-reported data (study 1), and a retrospective cohort study using health claims data (study 2).

In study 1, patients in orthopedic follow-up rehabilitation after hip or knee arthroplasty and their physicians are repeatedly surveyed with a set of validated instruments at different phases throughout their post-op treatment. The instruments used allow comprehensive assessment of the various (long-term) outcome domains of rehabilitation (PD outcome variables), and are the current best-practice assessment against which the claims data outcomes (CD outcomes) will be tested (see section “Study outcomes”). Facility-level quality indicators based on primary data (PD quality indicators) will be calculated for individual facilities by grouped scale means.

**Fig. 1:**
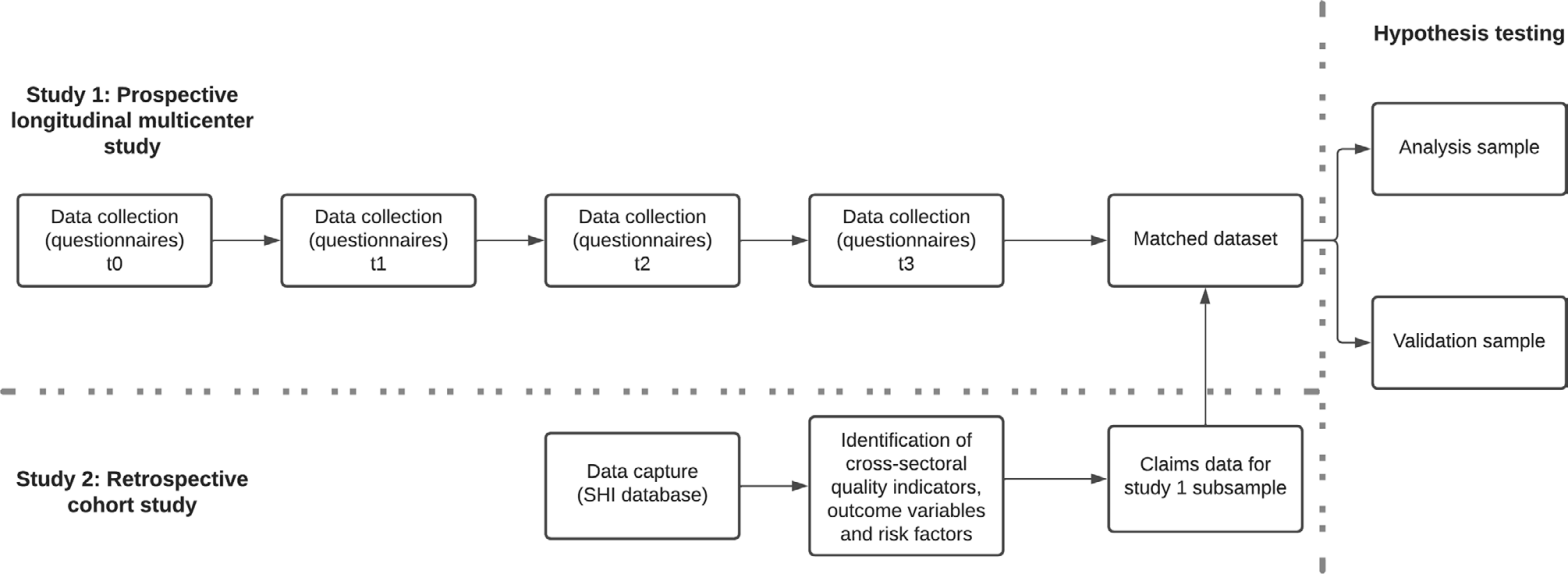
Data flow in SEQUAR

For study 2, health claims data (CD) will be extracted from the AOK health insurance fund database (see section “Sample”). Participants of study 1 are a small subsample of the larger study 2 sample. The large study 2 sample will be used to explore, identify and validate potential CD outcome variables, CD quality indicators, and CD risk factors. The subsample of claims data from study 1 participants will be matched to the outcome variables in the PD dataset resulting from study 1, and split into two subsamples for analysis and validation, respectively. Figure 1 illustrates the data flow.

We will use the matched dataset to test our hypotheses:

For H1a, CD outcome variables should be able to serve as surrogates (proxies) for rehabilitation treatment outcomes as measured by current best-practice assessment (PD outcome variables; see outcome assessment and study design in high-quality studies on the effectiveness of multidisciplinary orthopedic rehabilitation—e.g., [28]). For H1b, we assume at least a moderate correlation (> 0.30) between CD quality indicators and PD quality indicators. To test H2, the impact of cross-sectoral CD risk factors (on patient and facility levels) on current risk-adjustment models for quality assessment will be investigated.

### Study population and recruitment

#### Study 1: prospective longitudinal study

For study 1, ten German inpatient rehabilitation facilities (IRFs) were enlisted in the study by local representatives of the regional AOK health insurance funds, which in turn are recruiting a planned sample of 600 patients. Participating IRFs signed a collaboration agreement with the principal investigator, and each appointed a local study coordinator in charge of organization and implementation of all project tasks (e.g., recruitment of participants, distribution of patient information and consent forms, storing/safekeeping signed consent forms). Patient recruitment began in November of 2022 and will end in December 2023.

Patients eligible for participation are recruited on-site in the beginning of their orthopedic follow-up rehabilitation. Study coordinators present them with a physical copy of the study fact sheet (covering study synopsis, data protection, and other legal aspects), and ask for their participation. Those agreeing to participate sign an informed consent form. Eligibility is determined by meeting the inclusion criteria: Participants must be at least 18 years old at time of inclusion (beginning of rehabilitation), insured through AOK health insurance fund (for purposes of data availability), and receive inpatient orthopedic follow-up rehabilitation after implantation of a knee joint replacement or hip joint replacement due to osteoarthritis. Participants need to demonstrate sufficient knowledge of the German language and sufficient cognitive abilities to answer the comprehensive questionnaires independently. Patients that prematurely discontinue rehabilitation, demonstrate insufficient German or cognitive skills, or change health insurance within the study period will be excluded.

Participation in the study is incentivized with a €50 gift voucher for patients that complete all four questionnaires, while IRFs receive €120 per patient for full patient participation and completed case report forms.

#### Study 2: retrospective cohort study

Included in the study 2 sample will be all AOK-insured patients that underwent primary hip or knee arthroplasty due to osteoarthritis and participated in orthopedic inpatient follow-up rehabilitation between 2018 and 2023. Cases are identified in the SHI claims database using the diagnoses and procedures described in Table 1, based on the respective yearly version of the International Classification of Diseases 10, German Modification [29] and the German Operation and Procedure Codes (OPS, [30]).

**Table 1:** Inclusion codes for retrospective cohort study

Procedures and diagnoses	Codes
Hip joint replacement	OPS: 5-820.0, 5-820.8, 5-820.9, 5-820.x
with discharge diagnoses	ICD-10: M05-M08, M16, M87
Knee joint replacement	OPS: 5-822.0, 5-822.g, 5-822.h, 5-822.j, 5-822.k
with discharge diagnoses	ICD-10: M05-M08, M17, M87

### Sample size

#### Study 1: prospective longitudinal study

**Fig. 2:**
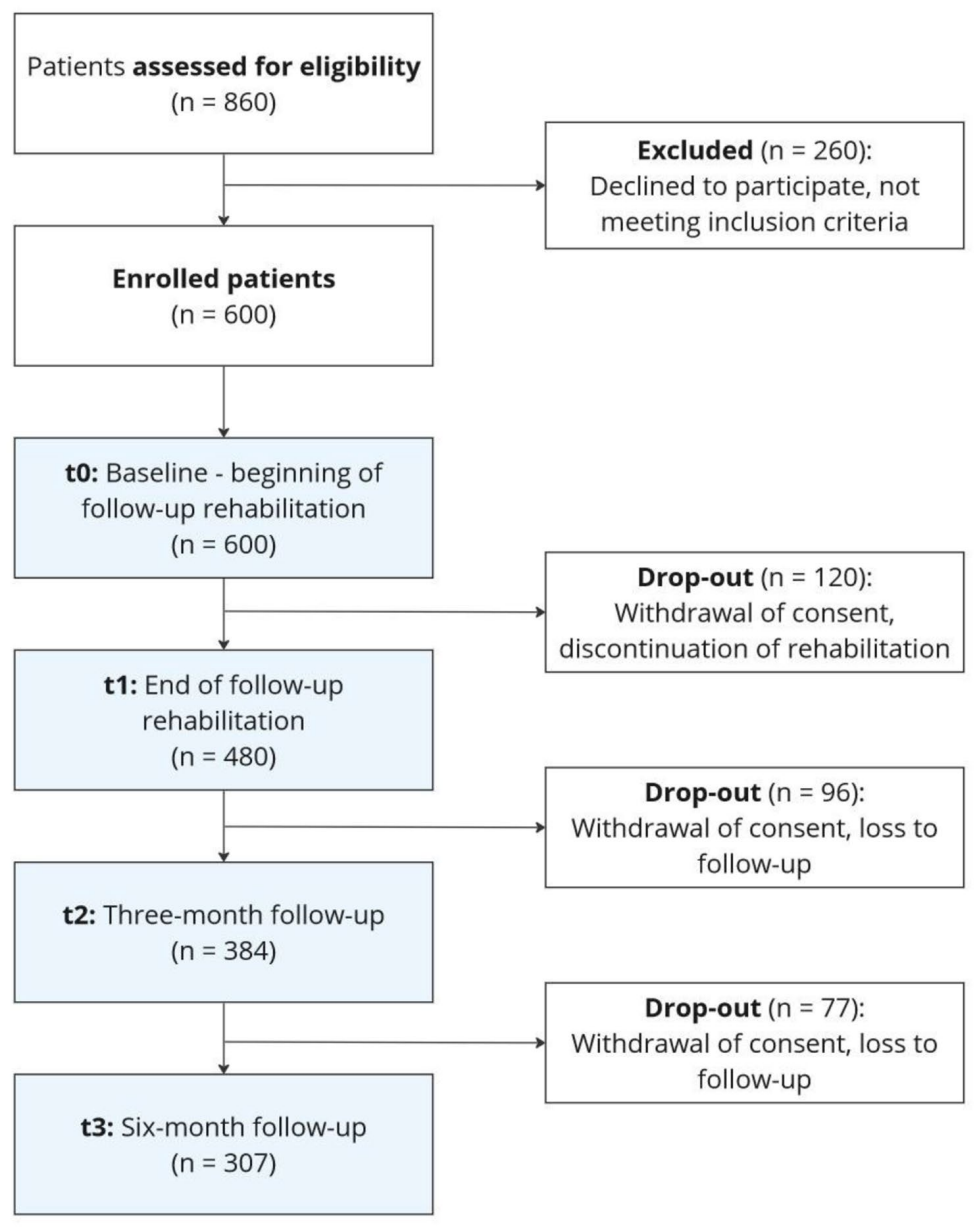
CONSORT flow diagram for study 1 (prospective longitudinal multicenter study)

Recruiting IRFs are encouraged to assess every patient for eligibility (as per the inclusion criteria), with a target sample of 60 participants per facility totaling *N* = 600 cases. Assuming an initial refusal quota of 30% (non-response), IRFs need to approach 860 patients, or 86 patients per facility. Withdrawal of consent or loss to follow-up throughout the data collection phase are estimated to result in an average dropout rate of 20% for each time point, which will reduce the initial target sample by approximately 50%. Thus, we expect a sample of n = 300 to remain for analysis (comparable studies report dropout rates of 25–55%, depending on the length of observational period; [31–33]). Figure 2 visualizes the sample flow in a CONSORT flow diagram.

#### Study 2: retrospective cohort study

Based on data from previous years, we expect around 27,000 patients nationwide participating in follow-up rehabilitation after knee joint replacement and 25,000 patients participating in follow-up rehabilitation after hip joint replacement, amounting to a study population of around 52,000 patients per year. Since study 2 includes patients from 2018 to 2023, the total study sample will be around 312,000 patients.

### Data collection

#### Study part 1: prospective longitudinal study

Data for the prospective multicenter study will be collected over a period of approximately 7 months with paper-pencil questionnaires. Patient-reported outcomes are recorded at four time-points: in the beginning of rehabilitation (baseline; t0), completion of rehabilitation (t1), three-month follow-up after completion (t2), and six-month follow-up after completion (t3). T0 and t1 patient questionnaires are to be distributed to patients a maximum of 2 days after inpatient admission and a maximum of 2 days before discharge by the local study coordinator. Patient questionnaires for t2 and t3 (follow-ups) are sent to patients’ residential addresses with prepaid return envelopes. IRF medical staff are to record additional medical data in a case report form at baseline (t0) and at completion of rehabilitation (t1).

#### Study part 2: retrospective cohort study

**Fig. 3:**
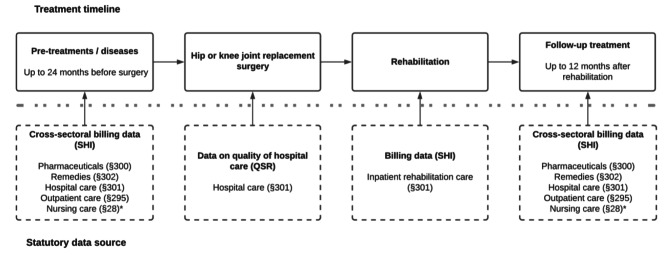
Data compiled for and used in the retrospective cohort study. *Note*: All sections cited are from the German Social Code Book V, unless they are marked with *, in which case the governing statute is the German Social Code Book XI

Claims data is sourced from the health claims databases of all 11 regional AOK health insurance funds. A project database will be compiled to include claims data from inpatient/hospital and outpatient care, medication, remedies and aids, as well as information on nursing care level and demographics. Additional data on the quality of the operating hospital is available through the established quality assurance procedure QSR [11]. The individual observation period for each patient includes 24 months before initial hospital admission and 12 months after follow-up rehabilitation. Figure 3 illustrates what data will be included in the project database and their statutory source in the German Social Code (see also section “Quality Assurance in German Rehabilitation”).

### Study outcomes

#### Study 1: prospective longitudinal study

For study 1, the various outcomes and outcome domains are measured with different instruments and collected from different sources, as displayed in Table 2.

**Table 2:** Outcomes, instruments, and data sources for study 1

Outcome (Domain)	Instrument	Data Source*	Time Point			
			t0	t1	t2	t3
Activities of daily living, emotional coping, mobility, physical functioning	Short Musculoskeletal Function Assessment (SMFA-D; [34])	P	x	x	x	x
Illness-related impairment/ “bother”	Short Musculoskeletal Impairment Assessment (SMFA-D; [34])	P	x	x	x	x
Physical pain	Visual Analogue Scale [35]	P	x	x	x	x
Pain-related self-efficacy	Pain Self-Efficacy Questionnaire (FESS; [36])	P	x	x	x	x
Walking ability	6 min Walk Test [37]	M	x	x		
Hip-specific outcome aspects such as limping, use of walkers, walking distance, climbing stairs, putting on shoes/socks	Harris Hip Score [38]	M	x	x		
Knee-specific outcome aspects such as mobility, stability	Knee Society Score [39]	M	x	x		
*Confounders*						
Comorbidity	Self-Administered Comorbidity Questionnaire (SCQ-D; [40])	P	x	x		
Age	Single item	P/M	x	x		
Gender	Single item	P/M	x	x		
Socio-economic status	Single item	P	x			
COVID-19-related stress	COVID-19 Stress Screening**	P	x			
Comorbidity	KOMO Score [41]	M	x			
Main and secondary diagnoses	ICD-10-GM codes [29]	M	x			

*P = patient-reported data, M = medical staff-reported data. **This instrument was developed by the principal investigator in 2020, publication is in progress. A translation of the adapted screening is attached in Additional File 1

#### Study 2: retrospective cohort study

Since study 2 is exploratory in nature, no CD outcomes have yet been identified. The following steps will be taken for selection and operationalization of CD outcome variables, CD quality indicators, and CD risk factors:

1. A structured search for existing quality indicators, outcome variables, and risk factors of orthopedic rehabilitation will be conducted in (a) literature (e.g., Medline, Cochrane Library), (b) medical guidelines (Guidelines International Network), (c) indicator databases (e.g., [42, 43], and (d) documentation of existing procedures of quality assurance in the field of orthopedic rehabilitation. Search results will be assessed for their compatibility with claims data.
2. The nationwide project claims database of AOK-insured patients will be analyzed to identify additional complex healthcare and rehabilitation outcomes for patients that have undergone primary knee or hip arthroplasty. This includes analysis of demographics, medical and other relevant data such as comorbidities, nursing care level, medication use, and utilization of therapeutic health service. The database will encompass data from the individual observational period for each patient, which includes the 24 months pre-surgery as well as 12 months of care after orthopedic rehabilitation. Pre-surgery data will be used as a baseline measure of study population characteristics, while the follow-up period after rehabilitation can be used to identify individual changes in relation to the baseline.
3. Results from step (1) and (2) will be consolidated and CD outcomes and risk factors operationalized. Potential CD outcomes that represent aspects of rehabilitation treatment quality within claims data could be, for example, mobility (through provision of mobility aids), pain medication, or progression to higher nursing care levels. CD outcomes will consider care and quality aspects from all medical sectors. All quality indicators will be defined by denominators and numerators, and descriptions of inclusion, exclusion and exception criteria [44]. Denominators display a specified target population (e.g., all patients undergoing arthroplasty), whereas numerators display the number of people in the target population experiencing a specified outcome (e.g., surgical complications; [44]). These descriptive definitions can later be used to calculate individual values for rehabilitation facilities. Indicators will be translated into coded data (e.g., coded diagnoses, procedures, and prescriptions), and an appropriate reference value will be defined for each indicator.
4. Each CD quality indicator will be adjusted for risk factors that have statistical influence on the indicator rate but cannot be influenced by IRFs. Risk factors might include patient characteristics (e.g., age, sex, comorbidity, nursing care level), or aspects of acute hospital care before rehabilitation. Relevant risk factors will be selected based on current research, results from SHI data analyses, and expert opinion [45].

### Data analysis and hypothesis testing

#### Study 1: prospective longitudinal study

Missing values analysis will be performed for each variable. If the results support the assumption of values missing at random or completely at random, they will be imputed via multiple imputation. Within the framework of sensitivity analysis, the hypotheses will be tested with both the raw dataset and the imputed dataset.

##### Hypothesis 1

To test hypothesis H1, the PD outcome variable dataset collected in study 1 will be matched to the CD outcome variable dataset using participants’ national health insurance numbers, which are pseudonymized by a data trustee in accordance with German data protection laws. Three dimensions of diagnostic validation will be evaluated to assess whether the CD variables can serve as proxies for the PD variables (see section “Study outcomes”): accuracy, reproducibility, and precision [46].

Accuracy will be assessed by linear regression, with each PD outcome variable as a dependent and each CD outcome variable as an independent variable. 95% confidence intervals (CIs) will be calculated for the identified intercepts and regression coefficients using the Gaussian approach (95%-CI of a: a ± 2*Se_a_; 95%-CI of b: b ± 2*SE_b_). If the CIs include 0 and 1 (for intercepts and regression coefficients, respectively), accuracy will be considered adequate for the outcome variable.

Reproducibility will be assessed by duplicate standard deviation (SD), which is calculated using the following formula (with *d* as the individual difference between the CD outcome variable and approximated PD outcome variable for each patient):

$$\varvec{D}\varvec{u}\varvec{p}\varvec{l}\varvec{i}\varvec{c}\varvec{a}\varvec{t}\varvec{e} \varvec{S}\varvec{D}=\sqrt{1/2\left(\frac{\sum {\varvec{d}}^{2}}{\varvec{n}}\right)}$$

Following the recommendation by Cleophas & Zwinderman [46], reproducibility is considered adequate for a duplicate SD of 10–20%.

Precision will be assessed by comparing the standard deviations of CD outcome variable and approximated PD outcome variable. If the standard deviation of a CD outcome variable is equal or smaller to the standard deviation of an approximated PD outcome variable, precision is considered good or better.

If at least two of the three validation criteria are met, the tested CD outcome variable is considered an adequate proxy for the respective PD outcome variable. Bland-Altman plots will be used for graphic evaluation of measurement differences [47, 48]. This same procedure will be applied to test H1b using CD quality indicators and PD quality indicators, which are represented by facility group mean values of PD outcome variables.

##### Hypothesis 2

H2 postulates an improvement in risk-adjustment models when considering cross-sectoral claims data, i.e., an increase in (adjusted) R^2^, and will be analyzed with nested multilevel regression models. A basic model (M0) will be estimated for each PD outcome variable using the PD confounders (see Table 2) on level 1. Models will be estimated for each time point after baseline (t1, t2, t3), and will be adjusted for *time* (rehabilitation effect) as a predictor on level 2. Furthermore, level 2 PD quality indicators will be included. Each model will be expanded with previously identified CD risk factors (on level 1 and level 2) as described in section “Study outcomes”. The nested models will be compared with Likelihood Ratio tests [49]. The analysis sample will be randomly divided into two parts, with one half allocated for analysis of risk-adjustment, and the remaining half used to validate the resulting models.

#### Study part 2: retrospective cohort study

Data of patients who switch health insurance or decease within their individual observation period will be censored from claims data.

The claims database with be analyzed with descriptive statistics, more precisely summary statistics (measures of central tendency, spread, and dispersion), and frequency distributions. We use stratified analyses and multivariate modelling (logistic regression and generalized linear models) to estimate the influence of potential risk factors. The data structure requires multi-level regression models for cluster effects: On a patient level, we estimate the influence of case-mix variables (e.g., age, sex, comorbidity, nursing care level) on each specified outcome within a potential CD quality indicator (see section “Study outcomes”). On a facility level, we estimate the influence of quality of acute hospital care on each specified outcome within a potential CD quality indicator.

The existence of further cluster effects at IRF level will be explored. CD quality indicators will only be adjusted for risk factors an IRF has no control over, e.g., a patient’s age. Relevant measures to assess goodness-of-fit for logistic regression models are Hosmer-Lemeshow-test, Receiver Operating Characteristic (ROC Curve), Area under the curve (ROC AUC) and pseudo-R2 [50]. For general linear models, a robust estimator for estimation of covariance matrix is used.

## Discussion

Health claims data has the potential to simplify current quality assessments in rehabilitation. SEQUAR will evaluate its benefit to quality assurance in orthopedic inpatient rehabilitation: We will explore outcome variables and quality indicators based on readily available statutory health insurance (SHI) claims data, and assess their ability to replace select parts of the current best-practice quality assurance protocol based on arduous primary data collection. If we fail to identify suitable claims data quality indicators, we can demonstrate that the current protocol of assessing patient-reported and physician-reported outcomes via questionnaires is still to be considered the best-practice approach. In the process, we are hoping to improve risk-adjustment models used to evaluate inpatient rehabilitation facility (IRF) quality by incorporating cross-sectoral SHI quality data (i.e., quality of the operating hospital) that could help adjust for risks outside of an IRF’s control.

Many previous studies have explored the utility of claims data in quality of care research. Some have even taken a cross-sectoral approach, like Stegbauer et al. [51] or Bramesfeld et al. [52], though the latter had to cut their trial phase short because–fittingly–the burden of primary data collection for participating physicians proved to be too great. However, to our knowledge, no study has examined claims data’s cross-sectoral potential for quality assurance in inpatient rehabilitation (where quality assessment is particularly difficult due to the variety in outcomes), nor has any research explored the possibility of replacing patient- and physician-reported outcomes with outcomes based on claims data.

If successful, further research is required to examine whether our findings are applicable to other indications of rehabilitation. SEQUAR focuses on a sample of post-op arthroplasty patients, since the underlying disease (gonarthrosis/coxarthrosis) and its degenerative effect is localized to a specific joint, diagnosed with an objective set of criteria, and treated with a limited number of specific surgical interventions [53–56]. Arthrosis primarily affects objective areas of functioning (such as walking, using stairs, doing yard work), and its subsequent rehabilitation program has an unambiguous primary outcome goal: to restore (painless) mobility [55, 56]. Objective impairment–as opposed to impairment that can only be measured via introspection, like symptoms of depression–can most likely be more easily described using (mostly objective) claims data. Other rehabilitation indications differ in their therapeutic programs and have other superordinate goals: Neurological rehabilitation after stroke seeks to restore motor function and strengthen adaptive strategies [57]; oncological rehabilitation focuses on coping and other psychosocial outcomes [58]; and cardiovascular rehabilitation targets lifestyle risk factors (e.g., nutrition, smoking, and lack of movement; [59]). If we identify an indicator that can reliably represent a more generic outcome of rehabilitation (e.g., restoring or improving strength, endurance, or flexibility), it should transfer to other indications. The more introspective and (strictly speaking) non-medical the outcome (e.g., nutrition, or psychosocial aspects), the harder it will be to approximate with health claims data.

### Practical implications

The ultimate goal of SEQUAR is a novel, state-of-the-art quality assurance protocol for orthopedic rehabilitation that incorporates identified claims data quality indicators and reduces the amount of patient- and physician-reported primary data required. The protocol will be developed in collaboration with nursing and medical staff from participating IRFs. IRFs will receive individual report cards with key quality indicators based on our results to identify and remedy internal quality management issues.

### Strengths

SEQUAR’s design of linking claims data-determined outcomes with primary data-determined outcomes (using the current best-practice protocol) allows for evaluation of proximity of the two on an individual patient level. The claims database in SEQUAR is very large, nationwide, and cross-sectoral—and reusing readily available, existing data not only in our research, but also in quality assessments nationwide, could result in significant reduction of required resources (e.g., paper questionnaires, servers to store primary datasets, financial burden on IRFs).

### Limitations

While the claims database used in SEQUAR is large, it is sourced from only 11 (of the over 90 existing) statutory health insurers in Germany, whose members make up only about 37% of SHI insured Germans. The identification process of claims data outcomes will not be universal, as each SHI has a distinct structure to their claims databases.

Primary data collection also comes with a known set of challenges: Longitudinal studies frequently struggle with dropout at follow-up, particularly 6 months after the fact [31–33]. Since IRFs and patients volunteer their participation, there will be some self-selection bias in our sample. Additionally, regional AOK representatives pre-selected IRFs that were known to be cooperative and interested in quality management research, which results in further selection bias. Participants that decease during the study period are censored from data analysis, leading to a potential underestimation of negative outcomes and inappropriate fit of our models for those patients with worse health at baseline.

### Electronic supplementary material

Below is the link to the electronic supplementary material.

Supplementary Material 1

## Data Availability

All project-specific datasets containing German statutory health insurance data will not be publicly available due to German privacy laws. Researchers may obtain access to these datasets by submitting a formal inquiry to the appropriate data protection authority, pursuant to Sect. 287 of the German Social Code Book V. More information can be found in the SEQUAR data protection policy, which is available from the corresponding author upon request. The primary data collected in the prospective longitudinal multicenter study will not publicly available due to SEQUAR’s data protection policy in accordance with the European General Data Protection Regulation. Although disclosure of anonymized data upon reasonable request is possible, it may be restricted or limited by the European General Data Protection Regulation and German data protection laws, as well as the expenses of legally sound anonymization.

## Footnotes

1 Expenditures are cited for 2019 because the COVID pandemic and lockdowns affected the German medical rehabilitation sector greatly in 2020 and 2021. As of drafting this manuscript, numbers for 2022 are not yet available.

## List of abbreviations

WHO: *World Health Organization*
ICF: *International Classification of Functioning, Disability and Health*
IRF: *Inpatient Rehabilitation Facilities*
SHI: *Statutory Health Insurance*
AOK: *General Local Health Insurance Fund (German*:*Allgemeine Ortskrankenkasse)*
CD: *Claims Data*
PD: *Primary Data*
CI: *Confidence Interval*
ROC: *Receiver Operating Characteristic*
