# Supplementary material for: A cross-sectoral approach to utilizing health claims data for quality assurance in medical rehabilitation: study protocol of a combined prospective longitudinal and retrospective cohort study
Source: BMC Health Serv Res. 2023 Oct 17;23:1110. doi: 10.1186/s12913-023-10074-w (PMC10583441; doi:10.1186/s12913-023-10074-w)
Supplement: Supplementary file 1 — Supplementary Material 1 [file 12913_2023_10074_MOESM1_ESM.docx]

| **How much stress have you experienced due to the coronavirus pandemic (COVID-19) starting in 2020?** | | | | | | | | |
| --- | --- | --- | --- | --- | --- | --- | --- | --- |
|  |  |  | no stress at all | a little stress | moderate stress | a lot of stress | enormous stress |  |
|  | Stress related to your current **employment situation** |  |  |  |  |  |  |  |
|  | Stress related to your current **financial situation** |  |  |  |  |  |  |  |
|  | Stress related to your current state of **mental health** |  |  |  |  |  |  |  |
|  | Stress related to your current state of **physical health** |  |  |  |  |  |  |  |
|  |  |  | 1 | 2 | 3 | 4 | 5 |  |

**Additional File 1:** COVID-19-Screener

* *Note*: The original COVID-19-Screener was developed in German by Farin-Glattacker et al. (2020, publication in progress) and adapted for the SEQUAR questionnaires – this English translation has not been validated or used in our research, and is intended for informational purposes only.
